# Microbial adhesion and ultrastructure from the single-molecule to the single-cell levels by Atomic Force Microscopy

**DOI:** 10.1016/j.tcsw.2019.100031

**Published:** 2019-08-30

**Authors:** Audrey Beaussart, Sofiane El-Kirat-Chatel

**Affiliations:** a Université de Lorraine, CNRS, LIEC, F-54000 Nancy, France; b Université de Lorraine, CNRS, LCPME, F-54000 Nancy, France

**Keywords:** Atomic force microscopy, Single-molecule force spectroscopy, Single-cell force spectroscopy, Interaction, Adhesion, Tip functionalization

## Abstract

In the last decades, atomic force microscopy (AFM) has evolved towards an accurate and lasting tool to study the surface of living cells in physiological conditions. Through imaging, single-molecule force spectroscopy and single-cell force spectroscopy modes, AFM allows to decipher at multiple scales the morphology and the molecular interactions taking place at the cell surface. Applied to microbiology, these approaches have been used to elucidate biophysical properties of biomolecules and to directly link the molecular structures to their function. In this review, we describe the main methods developed for AFM-based microbial surface analysis that we illustrate with examples of molecular mechanisms unravelled with unprecedented resolution.

## 1 Introduction

The principle of atomic force microscopy (AFM) is to scan the sample surface with a nanometric tip mounted on a flexible cantilever on which a laser beam is focused and reflected in a photodiode that records cantilever deflections while sample scanning. The precise positioning of the tip over the sample is ensured by piezoelectric scanners working in x, y and z directions. This technique allows to image samples and to sense forces with subnanometer resolution and piconewton sensitivity, respectively. Since its invention in 1986 (Binnig et al., 1986), AFM has acquired numerous modes and options (*e.g.* tapping, contact and -more recently- multiparametric modes) that offer now the possibility to analyse biological samples in physiological conditions and to capture events in real time.

Applied to microbiology, AFM has opened new doors for the description of topographical features, cell wall associated molecular mechanisms and whole cell adhesion. This has been possible thanks to the development of new AFM modes and new methods that allow to use molecules or cells as probes during AFM measurements. Cell surfaces play crucial role in microbiology as they represent the direct interfaces between the cell and the external stimuli, signals and stresses from the environment. Thus biological functions are directly linked to the microbial surface composition and organization. Bacteria are divided in two major groups that both contain a peptidoglycan layer, *i.e.* glycan chains linked by peptide chains. Gram positive bacterial cell walls are covered by anionic polymers such as techoic acids whereas in Gram negative bacteria the peptidoglycan layer is thinner and surrounded by two phospholipids membranes (inner and outer), the outer one being decorated by lipopolysaccharides. Many bacteria are covered also by additional appendages involved in cell displacement, adhesion and exchanges with the surrounding environment (*e.g.,* flagella, fimbriae, pili). The cell wall of filamentous fungi and yeast cells is also involved in cell mechanical strength and environmental signalling. The general fungal cell wall architecture is made of fibrils of chitin and β-1,3-glucans that are overlaid by β-1,6-glucans and mannoproteins. Here, we review some of the major advances in the comprehension of microbial cell surface organization obtained thanks to AFM.

## 2 Imaging microbial surfaces and appendages

### 2.1 Immobilization methods

The first attempt in using AFM to characterize biological samples is usually to obtain high resolution topographic images. As surfaces of microbes are generally rigid and smooth, images with ~10 nm resolution can be easily obtained (Dufrene et al., 2017). As microbes are mostly round shaped, an often critical step for their imaging is the proper cell immobilization to avoid to push the cell with the tip instead of scanning the cell surface. The most commonly-used immobilization methodologies can be divided in three groups: i) embedding of part of the cell volume in gelatin (Doktycz et al., 2003, Beckmann et al., 2006, Allison et al.,, Van Der Hofstadt et al., 2015); ii) electrostatic immobilization on positively charged substrates (da Silva and Teschke, 2003, Jacquot et al., 2014); and iii) mechanical trapping in porous membranes (Kasas and Ikai, 1995, Dufrene, 2015). However, all these techniques present limitations and should be wisely chosen depending on the applications. Chemical substrate modifications (i or ii) are often selected when one wants to observe bacterial growth for instance (Van Der Hofstadt et al., 2015). However, gelatin substrates are not recommended as they can cause AFM tip contamination. Charged surfaces are often obtained by simple immersion of a glass or silicon substrates in polyethylenimine or poly-L-lysine, resulting in a positively-charged coating favouring the adhesion of negatively-charged microbial cell-walls. Such technique has been successfully used for bacteria (da Silva and Teschke, 2003, Vadillo-Rodriguez et al., 2008, Jacquot et al., 2014) and yeast (Arfsten et al., 2010). Microorganisms have also been deposited on polyelectrolytes multilayer coatings by centrifugation, enabling the observation of the proteinaceous surface layer at the bacterial surfaces (Gunther et al., 2014). Although these methodologies are appropriate for imaging and nanomechanical mapping, some charged polymer may alter cell viability or denaturate molecules grafted on tips for molecular mapping (see below) (Colville et al., 2010, Krapf et al., 2016).

**Fig. 1:**
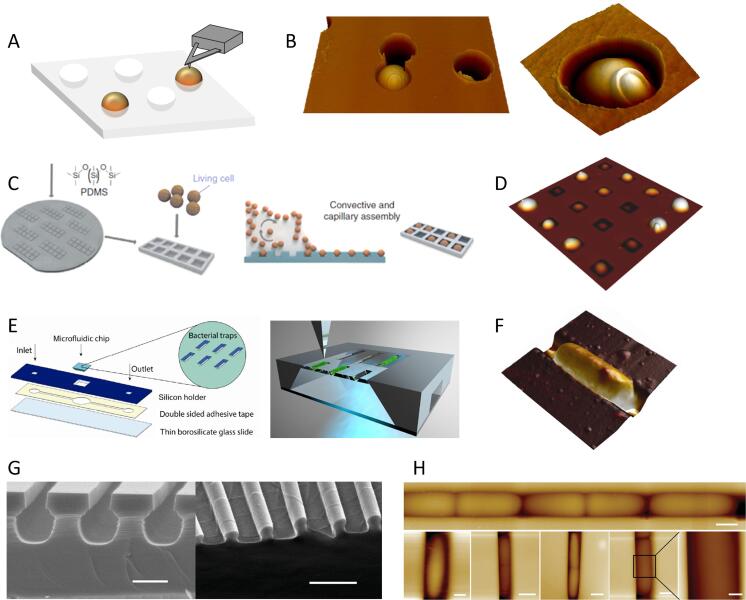
Different methods for proper physical entrapment of microorganisms. (A) Microorganisms suspensions can be filtered through a porous membrane whose pore size corresponds to that of the cells. (B) AFM deflection images of the yeast *Candida albicans* trapped in a pore, allowing high resolution images where a cell bud scar is visible. (C) Schematics of the immobilisation methods using PDMS microstructured stamps as developed by Formosa et al. (2015a). Living cells are assembled into the stamps using convective and capillary assembly. (D) AFM images of such PDMS cells arrays filled with *C. albicans.* (E) Immobilization in microfluidic device as developed by Peric et al. (2017). The microfluidic chip with bacterial traps is mounted to a square opening in the silicon holder. The underside of the device is transparent to allow simultaneous AFM and optical microscopy measurements. (F) AFM image of *Escherichia coli* bacterium trapped in such trap. (G) Immobilization in micro-tube arrays with an open-up structure as developed by Chen et al. (2014). SEM Images of the mold use to create PDMS micro-tubes structures. (H) AFM imaging of the bacteria dividing along the micro-channel. The design of the set-up allows for simultaneous AFM - fluorescent imaging. Fig. 1B, right, has been reproduced from (Dufrene, 2015) with permission from Elsevier Reprints. Fig. 1C-D have been reproduced from (Formosa et al., 2015a) with permission from Springer Nature. Fig. 1E, F have been reproduced from (Peric et al., 2017) with permission from Springer Nature. Fig. 1 G-H have been reproduced from (Chen et al., 2014) with permission from John Wiley and Sons.

Mechanical trapping in pores (Fig. 1A, B) is particularly adapted for high resolution imaging and molecular mapping but it is sometimes time consuming to find the good immobilization procedure and to determine the appropriate pore size, especially in the case of rod-shape bacteria. Physical entrapment may also have an influence on the mechanical properties of the cells, and/or may select a particular phenotype (of a given size) among the microbial population.

Development of the new AFM modes (*e.g.* Quantitative Imaging (JPK) or Peak Force Tapping (Bruker) modes) where the tip rapidly and punctually touches the sample has allowed to partially solve the immobilization problem, by limiting the lateral friction forces. As an example, bacteria under gliding movement as they were simply deposited on a glass substrate were observed by such technique (Dhahri et al., 2013). Nevertheless, such successful applications remain very scarce and usually work poorly in physiological conditions.

More recently, additional approaches based on the physical immobilisation of microbes have been developed (Fig. 1). Formosa *et al.* have elaborated polydimethylsiloxane (PDMS) stamps to immobilise arrays of living cells using a convective and capillary assembly without chemical or physical denaturation of the cells (Formosa et al., 2015a). This elegant protocol permits statistically relevant AFM measurements on several cells (Fig. 1C-D). Peric *et al.* have made use of microfluidics for pressure-driven anchoring of bacteria in V-shaped traps, where the lateral forces of the AFM tip during scanning are counteracted by the inclined walls (Peric et al., 2017). With this set-up, bacteria can be sequentially immobilized and released from the trap, and the transparency of the device allows simultaneous AFM and fluorescent imaging (Fig. 1E-F). Using combined micro-/nano-fabrication and soft lithography, Chen *et al.* designed arrays of micro-channels made of two-layers: a micro-tube for cell growth and a submicron opening at the top of the tube that provides access for the AFM tip (Chen et al., 2014). Thus, they managed to visualize *Escherichia coli* cell growth without chemical immobilization in such structures (Fig. 1 G-H).

### 2.2 Ultrastructural changes at the surface of cells

**Fig. 2:**
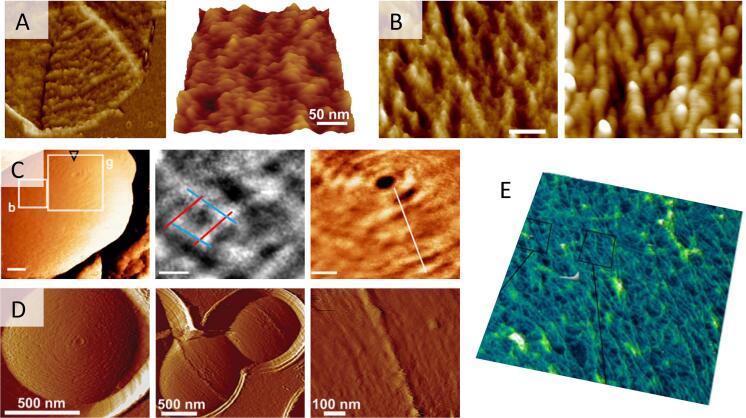
High resolution imaging of peptidoglycan. (A) Deflection images of sacculi from the Gram positive bacteria *Bacillus subtilis* (left) (Hayhurst et al., 2008) and *Lactococcus lactis* (right) (Andre et al., 2010). (B) Different structural organisations observed on the side wall peptidoglycan from *B. subtilis* in mid-exponential phase (left) compared to that in stationary phase (right) (Li et al., 2018). (C) High resolution images taken on living Group B *Streptococcus* reveal a nanoscale net-like surface architecture (left, middle), and circular arrangement of bands around the pole (right) (Dover et al., 2015). (D) Images of *L. lactis* living cells also show circular arrangement of the peptidoglycan at the bacterial pole (left) and periodic bands running parallel to the short cell axis on the bacteria longitudinal side (middle, right) (Andre et al., 2010). (E) Direct visualisation of glycan strand arrangement in the Gram negative bacteria *E. coli* envelope, obtained by mounting peptidoglycan fragments on poly-l-ornithine (Turner et al., 2018). Fig. 2A has been reproduced from (Andre et al., 2010) and (Hayhurst et al., 2008) with permission from Springer Nature and the National Academy of Sciences. Fig. 2B has been reproduced from (Li et al., 2018) with permission from Frontiers. Fig. 2C has been reproduced from (Dover et al., 2015) with permission from Springer Nature. Fig. 2D has been reproduced from (Andre et al., 2010) with permission from Springer Nature. Fig. 2E has been reproduced from (Turner et al., 2018) with permission from Springer Nature.

With all these immobilization methods, AFM has been successful to unravel microbial morphological features at high resolution. One notable example is the study of the spatial arrangement of peptidoglycan, a fundamental structural constituent of the bacterial cell wall. High-resolution architecture of peptidoglycan was first observed on purified sacculi from *Bacillus subtilis* (Hayhurst et al., 2008) (Fig. 2A) before being imaged directly on living *Lactococcus lactis* (Andre et al., 2010) (Fig. 2D). Both studies agree that peptidoglycan is organized -at least on Gram-positive bacteria- into a regular structure of cables with cross striations perpendicularly to the long cell axis. A more recent analysis by Li *et al*. revealed that the peptidoglycan would be subjected to a remodelling during the growth of the bacteria, which would change from an irregular architecture in exponential growth phase to an ordered cable-like architecture in stationary phase (Li et al., 2018) (Fig. 2B). Dover *et al.* also demonstrated how the structure and elasticity of the peptidoglycan of Group B *Streptococcus* change when subjected to increasing turgor pressure (Dover et al., 2015) (Fig. 2C). Peptidoglycan organisation has also been deciphered very recently on sacculi of the Gram-negative bacteria *E. coli* (Turner et al., 2018) (Fig. 2E). In this study, Turner *et al.* quantified and mapped the extent to which the glycan chains are oriented in a similar direction (orientational order), and showed that it is much less ordered than previously depicted. Bacterial imaging has also allowed Eskandarian *et al.* to decipher the mechanisms of cell division (Eskandarian et al., 2017). In mycobacteria, septum formation and division would occur within wave troughs on the undulating cell surface, which might be directly related to the peptidoglycan architecture underneath.

On fungal spores, AFM has been used to image the nanorods that protect *Aspergillus fumigatus* spores (Bayry et al., 2014). As AFM works in liquid and can therefore adapts to cell physiological conditions, Dague *et al.* used time-resolved-imaging to capture *in situ* the disorganization of *A. fumigatus* spores nanorods under germination upon temperature variation (Dague et al., 2008). Similarly, alteration of the spore coat architecture during germination process has been revealed on *Bacillus atrophaeus* (Plomp et al., 2007), a destructuring which is also associated with changes in the elasticity of the spores (Pinzon-Arango et al., 2010).

AFM in liquid conditions has also permitted the direct observation of microbial cells subjected to external chemical agents. In that sense, *Staphylococcus aureus* cell wall digestion has been followed upon injection of the enzyme lysostaphin in the AFM liquid cell while continuous scanning**.** Following the drug injection, the authors observed the bacterial swelling, splitting of the septum and the formation of holes that they attributed to the peptidoglycan digestion by the lysostaphin (Francius et al., 2008). Then Formosa *et al.* have focused on the resistance of the bacteria *Pseudomonas aeruginosa* under the effect of two antibiotics and an innovative antibacterial drug (CX1) by recording the drug effects on the morphology, the roughness and the nanomechanical properties of the cell. As such, they demonstrated for instance that the cell outer membrane get destroyed under the drug CX1 action (Formosa et al., 2012). The disrupting morpho-structural effects induced by rokitamycin and erythromycin on *Streptococcus pyogenes* has also been reported, evidencing important differences between the two drugs mode of action. Whereas bacteria do not visually get impacted by erythromycin, the cells subjected to rokitamycin get deformed, loose their chain structure and form cell clusters (Braga et al., 2002). With the same perspective, Fantner *et al.* have investigated the kinetics of individual *E. coli* cell death under the action of the antimicrobial peptide using high-speed AFM, demonstrating that the killing is a two-phase-process (Fantner et al., 2010).

On yeast cells, several studies have shown the capabilities of AFM to image the cell wall at high resolution, revealing a smooth surface with sometimes budscars (Alsteens et al., 2010, Dupres et al., 2010, Dupres et al., 2011, El-Kirat-Chatel et al., 2013a) (Fig. 1B). *Candida albicans* is a pathogenic yeast that can form long germtubes. This morphological change impairs the mechanical-trapping immobilization process usually used for microbes. Beaussart *et al.* have then developed a method that relies on hydrophobic interactions to image the surface of *C. albicans* hyphae (Beaussart et al., 2012). Yeasts may be subjected to numerous stresses that are likely to affect cell growth and metabolic activity. As previously reported for bacteria, the effect of external stimuli can also change the smooth surface of yeast cells. For instance, after antifungal treatment (caspofungin), AFM imaging has revealed that yeast cell wall presented irregular and rough surface together with a decreased mechanical strength determined by nanoindentation (El-Kirat-Chatel et al., 2013a, Formosa et al., 2013, Quiles et al., 2017). The morphology and nanomechanical properties of *Saccharomyces cerevisiae* defective mutants have also been compared (Dague et al., 2010). Physico-chemical external stimuli, *e.g.* heat shock, also generate morphological aberrations such as formation of circular structures at the cell surface which have been attributed to a dysfunction of the yeast budding machinery under temperature changes (Pillet et al., 2014). The detrimental effect of ethanol -to which yeasts are exposed in bioethanol fermentation processes for instance- on *S. cerevisiae* has been extensively reported in terms of morphological and nano-mechanical modifications (Canetta et al., 2006, Niu et al., 2016, Schiavone et al., 2016).

Despite its capability to image biological samples in physiological conditions and at high resolution, observing labile structures such as *e.g.* flagella or pili on living cells remains a real challenge. This limitation has been overcome by imaging bacterial appendages of dried samples. Images of flagella immobilized on the substrate after drying have been obtained for several species such as *Pseudomonas fluorescens* (Diaz et al., 2011), *Bacillus thuringiensis* (Gillis et al., 2012a, Gillis et al., 2012b), and *E. coli* (Francius et al., 2011). All these images of flagella reveal structures that are longer than the bacterial cell body and that present regular curvature reflecting their flexibility. Smaller appendages that contribute to bacterial adhesion were also imaged in air, revealing the structures of pili and curli of *e.g. Lactobacillus rhamnosus* (Tripathi et al., 2012), *Pseudomonas aeruginosa* (Touhami et al., 2006, Beaussart et al., 2014a), *Corynebacterium diphtheria* (Rheinlaender et al., 2012) and *Salmonella* (Jonas et al., 2017).

Besides imaging, AFM can be used to detect and manipulate molecules at the surface of living cells with the so-called force spectroscopy mode. In this mode, the tip is constantly approached and retracted from the surface in the z direction to sense interaction forces that occur between the tip and the sample. This can be achieved in a defined area, thus resulting in mapping of the interaction forces across the sample. The next chapter describes some achievements made with AFM in single-molecule force spectroscopy (SMFS) mode for a better understanding of the molecular mechanisms taking place at the microbial surface.

## 3 Single-molecule mapping on microbial surfaces

### 3.1 Functionalization strategies resulting in a covalent linking of the biomolecules to the AFM probes

AFM is not only an imaging tool. Additionally to high resolution imaging, researchers often want to decipher structural/biophysical properties and to understand molecular mechanisms taking place at the cell surface. This can be achieved with AFM when tips are functionalized by attaching specific (bio)molecules at their apex and measuring the unbinding forces between the tip and the biosurface (Hinterdorfer and Dufrene, 2006). Several protocols developed to graft molecules on AFM tips have been used to probe microbial surfaces. Surface hydrophobicity is an important property for airborne spreading, adhesion and cellular contact. To determine -at the nanoscale- the hydrophobic balance of microbial surfaces, gold coated tips were immersed in solutions of thiols terminated with methyl groups to form a hydrophobic self-assembled monolayer (SAM) (Alsteens et al., 2007). With these hydrophobic tips, it has been possible to correlate the high hydrophobicity of *A. fumigatus* spores with the presence of nanorods (Dague et al., 2007, Dague et al., 2008, Alsteens et al., 2013a, Bayry et al., 2014). On *Candida* yeast cells, hydrophobic tips have been used to directly link surface hydrophobicity with the expression of i) agglutinin-like sequence proteins (Als) that present conserved hydrophobic domains on *C. albicans* (Beaussart et al., 2012) and ii) epithelial adhesin (Epa) on *C. glabrata* (El-Kirat-Chatel et al., 2015a), both proteins being involved in adhesion and cell aggregation. Yet, this approach with SAM-covered tips lacks in specificity as all molecules presenting hydrophobic domains can potentially interact with the tip. To assess specific receptor-ligand interactions, and understand the complex molecular association and dissociation processes involved in such recognition, AFM tips need to be decorated with specific biomolecules *e.g.* lectins, antibodies. Jauvert *et al.* have developed a method where tips are first functionalized with amino groups, then react with aldehyde-phosphorus dendrimers that serve to graft biomolecules containing free amino functions (Jauvert et al., 2012). Using this methodology to functionalize tips with the lectin Concanavalin A, Formosa *et al.* have highlighted how antibiotics induce molecular rearrangements in *Pseudomonas* cell wall, additionally to morphological and mechanical changes (Formosa et al., 2012). Another methodology to covalently attach biomolecules to the tip consists in immersing the probe in alkanethiols that terminate in carboxyl functions, which can then be reacted with the amino groups of proteins using 1-ethyl-3-(3-dimethylaminopropyl)carbodiimide (EDC) and N-hydroxysuccinimide (NHS) (Dammer et al., 1995, Grandbois et al., 2000, Herman-Bausier et al., 2018).

Another largely used method is the protocol developed in the group of Prof. H. Gruber, Linz, Austria. Here, polyethylene glycol (PEG) molecules react with amino-functionalized tips and present on their other extremity an aldehyde (or aldehyde precursor) group that will react with the amino groups of the biomolecules (Ebner et al., 2007, Ebner et al., 2008, Wildling et al., 2011). The elegance of this method resides in several points: i) the attachment procedure results in covalent binding of the molecule to the tip, which is then much stronger than the receptor-ligand force being studied (Grandbois et al., 1999), ii) the quantity of chemical reagents used ensures that very low density of molecules are grafted on the tip apex, allowing single-molecule interactions to be probed, iii) the PEG linker permits to maintain a certain mobility of the attached molecules which can then freely interact with their receptors, and iv) inert PEG linkers limit the probability of unspecific interactions with the biosurface components.

Tip functionalization through PEG linkers has been used to decipher the molecular organization of several microbial cell walls. For instance, in complement to the imaging previously described, the peptidoglycan structure and composition have been revealed by tips functionalized with lectins or peptidoglycan hydrolases (Andre et al., 2010, Beaussart et al., 2013c). This molecular mapping is highly accurate and can be nicely correlated with the detection of peptidoglycan cables observed on the topographic images of the cell wall as demonstrated on *L. lactis* (Andre et al., 2010) and *Streptococcus agalactiae* (Beaussart et al., 2014c). Additionally to the detection of structural components, tip functionalization can be used to detect and manipulate molecules involved in microbial adhesion or in the interaction with the host. Several examples demonstrate that SMFS is powerful to decipher the mechanical properties of bacterial appendages. Using tips decorated with pili sub-units, Tripathi *et al.* were able to specifically probe and unfold pili on the Gram-positive living bacteria *L. rhamnosus* GG (LGG) (Tripathi et al., 2013). The peculiar force signatures obtained presented single large adhesion force peaks with linear shape and characteristic horizontal force steps. These signatures reflect the nanospring mechanism by which pili withstand shear forces during bacterial adhesion. This was further confirmed with tip decorated with milk proteins, the target of LGG pili for adhesion in dairy (Guerin et al., 2018a, Guerin et al., 2018b). Very different force distance signatures were obtained for the unfoding of pili from Gram-negative bacteria such as *P. aeruginosa* and *E. coli* (Forero et al., 2006, Beaussart et al., 2014a, Mulansky et al., 2017), resulting in plateaus preceded by a region of zero force, which are most likely due to a force-induced conformational changes within individual pili.

**Fig. 3:**
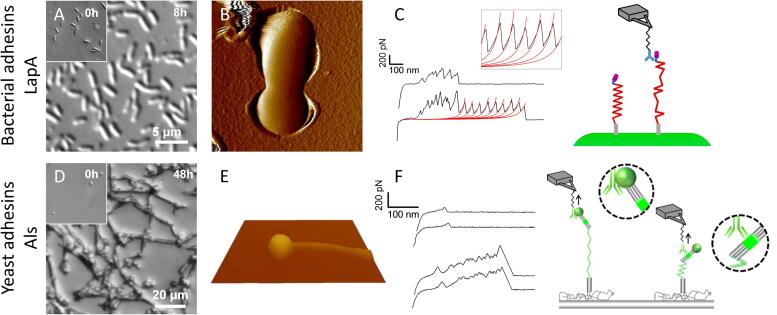
Similitude of adhesion mechanism through the expression of adhesins for the bacteria *Pseudomonas fluorescens* (A–C) and the pathogenic yeast *Candida albicans* (D–F). (A, D) Optical images (A: Phase, D: DIC) showing the microscopic adhesion of microorganisms to hydrophobic substrates after several hours of incubation. (B, E) AFM deflection images of single microorganisms immobilized by trapping in porous membrane or hydrophobic interactions. (C, F) Representative force-distance curves obtained during the unfolding of adhesins using functionalized antibody tips. Some curves represent single weak epitope recognition and others feature sawtooth patterns documenting repeated-regions unfolding. Fig. 3A–C have been reproduced from (El-Kirat-Chatel et al., 2014a) and Fig. 3D–F have been reproduced from (Beaussart et al., 2012) with permission from the American Chemical Society.

SMFS with tips functionalized with specific antibodies have also allowed to decipher the distribution and mechanical properties of various adhesins expressed at microbial surfaces. For instance, LapA*,* a major adhesin of *Pseudomonas fluorescens,* is able to promote cell adhesion on surfaces of highly different nature (hydrophobic or hydrophilic) (Hinsa et al., 2003, Duque et al., 2013) (Fig. 3A–C). It contains a cell wall anchoring domain, 37 repeated sequences and a C-terminal globular domain. Using SMFS, these repeats were unfolded under the external force exerted by the AFM tip retraction, which has led to specific force signatures presenting regular sawtooth patterns (El-Kirat-Chatel et al., 2014a) (Fig. 3C). Remarkably, the same functionalized tips have revealed bacterial footprints made of adhesins left on the surface after bacterial adhesion and detachment (El-Kirat-Chatel et al., 2014b). On *Staphylococcus epidermidis,* SMFS has been used with fibrinogen functionalized tips to decipher the mechanism of the SdrG adhesion (Herman et al., 2014). This work has shown that SdrG adhesin recognizes and adheres to human fibrinogen with a remarkable strength, similar to a covalent bond (*i.e.* > 2 nN), through a so called “dock, lock and latch” mechanism and therefore promotes bacterial infection and persistence on medical devices. The same authors demonstrate that such strong forces is a recurrent mechanism among *Staphylococcus* species, which adhere strongly *e.g.* to fibrinogen *via* the clumping factor A when subjected to mechanical tension (Herman-Bausier et al., 2018), to collagen *via* the Cna proteins through a ‘collagen hug mechanism’ (Herman-Bausier et al., 2016) and to collagen *via* SdrF proteins (Herman-Bausier and Dufrene, 2016). On yeast cells, the combination of different specific probes has allowed to determine the distribution and abundancy of different cell wall glycans -namely mannosides, glucans and chitin- in three different yeast species (El-Kirat-Chatel et al., 2013b). This work has led to the conclusion that glycans are differentially exposed on pathogenic yeast and are linked to longer polysaccharide chains which may interfere with the recognition by the host immune system. During infections, adhesion of *C. albicans* yeast cells is one important pathogenic factor and is governed by adhesins from the Als family (Dranginis et al., 2007, Hoyer and Cota, 2016). Similarly to LapA from *P. fluorescens*, Als contains tandem repeats that contribute to hydrophobic interactions and a globular head with an immunoglobulin-like region that promotes adhesion to host proteins (Otoo et al., 2008, Lipke et al., 2012). The mechanism by which Als control *C. albicans* adhesion has been elucidated by SMFS with AFM tips functionalized with immunoglobulin sequences for homotypic recognition (Alsteens et al., 2009), anti-V5 antibodies for epitope-tagged Als recognition (Alsteens et al., 2010), anti-Als antibodies (Beaussart et al., 2012) (Fig. 3D–F) or with a pentapeptide recognized as a host protein (El-Kirat-Chatel et al., 2013a). Consecutive molecular mapping of Als on live yeast cells have shown that adhesins are able to cluster to form adhesive patches once they are mechanically stimulated. This allows the cell to increase local adhesion strength during either cell-substrate or cell-cell interactions (Alsteens et al., 2010). The presence of such adhesins clusters has also been observed in living *C. albicans* depending on the budding stage of the cells (Formosa et al., 2015b). Adhesive patches have been reported as well at the surface of a specific strain of *S. cerevisiae*, and attributed – *via* completementary transcriptomic analyses- to the flocculin proteins *FLO11* (Schiavone et al., 2015). Thanks to the immobilization technique developed by Beaussart *et al.* for *C. albicans* after morphogenesis (Fig. 3E), SMFS has been used to demonstrate that this morphological switch comes together with an overexposure of Als proteins and mannosides on the hypha (Beaussart et al., 2012) (Fig. 3E, F). Another study using SMFS to probe Als on *C. albicans* has shown that these adhesins are not only involved in infection but are also overexpressed when cells are exposed to external stresses, *i.e.* treatment with the caspofungin antifungal drug. This overexpression of Als leads to cell aggregation to protect cells from the drug and limits its diffusion (Gregori et al., 2011, El-Kirat-Chatel et al., 2013a).

### 3.2 Functionalization strategy allowing orientation of the biomolecules on the probes

All functionalization methods presented above result in a covalent linking of the biomolecules to the AFM probes. Although covalent linkage is robust, these methods graft molecules randomly from any free amino-group and are not suitable for oriented linkage. When a site-directed attachment is needed, one can functionalize gold coated AFM tips with nitrilotriacetic acid (NTA) and use them to graft His-tagged proteins in an oriented manner (Kienberger et al., 2000). Yet, this grafting method is less robust as the interaction force between a single His-6 tag and one NTA group is ~150 pN (Kienberger et al., 2000, Berquand et al., 2005, Dupres et al., 2009b). Besides using NTA-tips to graft biomolecules and probe their ligands on cells (Dupres et al., 2009b), NTA-tips can be directly used to map His-tagged proteins at the surface of living cells. The role and mechanism of the yeast plasma membrane mechanosensor Wsc1 was elucidated with this approach (Schmitt et al., 2000, Heinisch et al., 2010). Yeast cells expressing His-tagged Wsc1 were probed with NTA-tips and the results showed that this mechanosensor behaves like a nanospring and forms clusters under external force stimulation. Moreover, this work has allowed to measure the thickness of the yeast cell wall, as a minimal length of ~110 nm was needed for Wsc1 to be detected at the cell surface with NTA-tips (Dupres et al., 2009a).

Molecular imaging through AFM-based SMFS with probes bearing specific biomolecules has been powerful over the last two decades to decipher the organization of microbial surfaces at the individual molecule level. Although this high resolution method is essential to understand the mechanisms governing molecular functions and organizations, this approach does not inform on how all surface components act together to promote cell adhesion. In this context, probing the interactions at the cell level became necessary. To fill this gap, force spectroscopy was recently adapted to single-cell measurements with the so-called single-cell force spectroscopy (SCFS). Originally described for mammalian cells (Helenius et al., 2008), methodologies for SCFS of microbial cells have been developed in the last decade (Potthoff et al., 2012, Alsteens et al., 2013b, Beaussart et al., 2013a, Beaussart et al., 2014b, Potthoff et al., 2015).

## 4 Probing microbial adhesion at the single-cell level

The use of AFM tips functionalized with specific probes gives access to a precise understanding of the biophysical properties of cell wall associated molecules and have offered the possibility to directly link the molecular structures with their function. Yet, this approach at high resolution is not adapted when one wants to characterize cell surface mechanisms at the whole cell level. AFM-based force spectroscopy can also be applied to force measurements at the whole cell level with the developed single-cell force spectroscopy mode (Benoit and Gaub, 2002, Helenius et al., 2008). In this mode, instead of having a tip on the cantilever probing the surface, tipless cantilevers are used to attach single cell and measure their interactions towards biotic or abiotic surfaces. This technique has been first developed for large mammalian cells in order to understand the adhesion mechanisms to the extracellular matrix (Helenius et al., 2008). In microbiology, SCFS of yeast cells has also been achieved with tipless cantilevers. As for mammalian cells, yeast cells can be attached on cantilevers coated with the lectin Concanavalin A that binds mannosides on the cell surface (Helenius et al., 2008, te Riet et al., 2017). Te Riet *et al.* used this methodology to probe single *C. albicans* towards the surface of dendritic cells to demonstrate the role of N-glycans in the recognition by the dendritic cell-specific ICAM-3-Grabbing Non-Integrin (DC-SIGN) receptors of the host (Te Riet et al., 2017). Another approach to attach cells is to coat cantilevers with polydopamine, a marine-inspired bioadhesive known to adhere on any type of surfaces without denaturing biological samples (Lee et al., 2007). With polydopamine coated cantilevers, El-Kirat-Chatel and Dufrêne measured the interaction forces between single *C. albicans* cells and the surface of the immune cells, *e.g.* macrophages. By injecting inhibitors in the AFM fluid cell during the measurement, they have highlighted the major role of mannose recognition in the initial steps leading to phagocytosis (El-Kirat-Chatel and Dufrene, 2016). Alsteens *et al.* have demonstrated the importance of *C. albicans* Als adhesins for adhesion to abiotic surfaces of different hydrophobicity and to fibronectin (Alsteens et al., 2013b) as well as for cell aggregation through homotypic interaction (Alsteens et al., 2007). Similarly, the role Flo adhesins involved in cell aggregation of *S. cerevisiae* have been deciphered by SCFS (El-Kirat-Chatel et al., 2015b, Chan et al., 2016). The importance of amyloid interactions during Flo-mediated aggregation was demonstrated through the injection of Thioflavin T, a marker of amyloid aggregation’ (Chan et al., 2016). SCFS has been used also to understand how Epa adhesins promote adhesion of the fungal pathogen to medical surfaces (El-Kirat-Chatel et al., 2015a). In this study, *C. glabrata* WT and mutant cells were probed towards hydrophobic or hydrophilic model surfaces and the results showed that Epa are mostly involved in the adhesion on hydrophobic substrates (El-Kirat-Chatel et al., 2015a). Using the same polydopamine grafting methodology, the filamentous fungi *A. fumigatus* was attached to the AFM cantilever under its dormant spore and germinated forms (Beaussart et al., 2015). The authors were then able to determine the importance of the newly discovered polysaccharide galactosaminogalactan (GAG) (Fontaine et al., 2011, Gravelat et al., 2013) in the adherence of the pathogen to a variety of substrates, including its biological target: the mammalian cells pneumocytes (Beaussart et al., 2015).

**Fig. 4:**
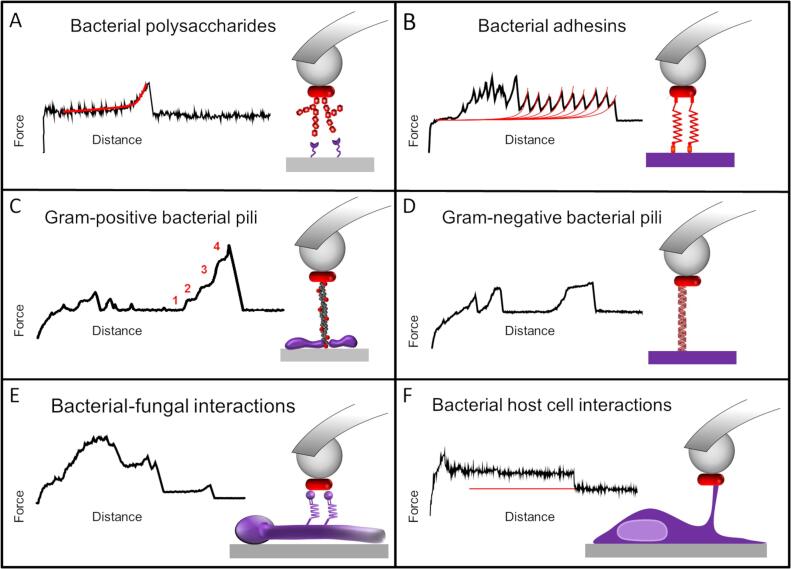
Single-cell force spectroscopy method to decipher bacterial adhesion mechanisms. The main surface components involved in the interactions are (A) bacterial polysaccharides, (B) adhesins, (C, D) pili, (E) multiple biomolecules in the case of two interacting microbes and (F) tethers formed by host cell membrane elongation in the case of host/microbes adhesion.

Concerning bacteria, tipless cantilevers coated with polydopamine or CellTak, a commercial wet cell adhesive, have been used to probe the interaction of *e.g. Staphylococcus xylosus*, *S. epidermidis*, *P. fluorescens*, *E. coli*, *L. plantarum*, *L. rhamnosus* GG or *Streptococcus mutans* towards different abiotic surfaces (Kang and Elimelech, 2009, Beaussart et al., 2013a, El-Kirat-Chatel et al., 2014a, Sullan et al., 2014, Zeng et al., 2014, Sullan et al., 2015). All these examples reveal the versatility of AFM-based SCFS to measure interactions between cells and substrates. Yet, as bacteria are small (usually at least one dimension < 1 µm), it has been stated that it is difficult to precisely position the cell at the apex of the cantilever and to avoid aspecific interactions coming from direct contact between the cantilever and the substrate (Beaussart et al., 2014b, Mulansky et al., 2017). To overcome this limitation, the preparation of colloidal probes where silica spheres are glued to tipless cantilever is a good alternative (Fig. 4). This approach allows a precise positioning of the bacteria and increases lifespan of the attached bacteria (Beaussart et al., 2014b, Mulansky et al., 2017). The proof of concept of this method was first obtained with *L. plantarum* cells probed towards hydrophobic or lectin-covered surfaces (Fig. 4A). In the context of mixed biofilms, this SCFS method adapted to bacteria has served to characterize the interaction between *S. epidermidis* and *C. albicans* hyphae (Beaussart et al., 2013b) (Fig. 4E). Combined to the SMFS analysis described, SCFS has been used to understand how different *Staphylococci* adhesins work at the whole cell level to promote adhesion to host proteins (Herman et al., 2014, Herman-Bausier and Dufrene, 2016, Herman-Bausier et al., 2016, Herman-Bausier et al., 2018). SCFS has been also essential to decipher the multipotent adhesiveness of LapA in *P. fluorescens* by probing several mutant strains towards surfaces presenting different chemical properties (El-Kirat-Chatel et al., 2014a) (Fig. 4B). It also allowed to better understand how pili from both Gram-positive (Sullan et al., 2014) and Gram-negative bacteria (Beaussart et al., 2014a, Beaussart et al., 2016) regulate bacterial adhesion to abiotic surfaces as well as mammalian cells (Fig. 4C, D).

Although SCFS presents several advantages as listed above, a main drawback of the technique is the lack of measurements that one can perform on different cells due to the time-consumption and complexity of the methodology. The Zambelli group, from ETH, Switzerland, has proposed an alternative by developing the so-called AFM-derived FluidFM, a method based on micro-nano-fluidics with hollow cantilevers to pick-up single cells by underpressure (Meister et al., 2009, Guillaume-Gentil et al., 2014, Amarouch et al., 2018). First developed with microchannels, it has been used for spatial manipulation of yeast by applying successively negative and positive pressures at the apex of the cantilever touching the cell surface (Dorig et al., 2010). The technique allows for high throughput screening as the same cantilever can be used successively to probe several microbes. In that sense, it has been used to quantify the adhesion of *C. albicans* towards hydrophobic surfaces and the authors were able to probe 200 cells with the same cantilever (Potthoff et al., 2012). The same principle was used to displace *E. coli* bacteria (Dorig et al., 2010). More recently, the development of cantilevers with nanochannels allows now to quantify the adhesion force of bacteria of different shapes and sizes, *e.g. E. coli* and *Streptococcus pyogenes* (Potthoff et al., 2015).

## 5 Conclusions

Since its initial development for material science, AFM has constantly evolved and allows now to analyse almost any biological sample in physiological conditions. As shown here, the capability of AFM to image living cells at high resolution and to sense small interaction forces has conducted to a precise description and understanding of the microbial cell surfaces topographies and their interface molecular mechanisms. Most recent AFM instruments can potentially be combined to optical/photonics microscopy tools. However, the development of correlated instruments (*e.g.* AFM coupled to single-molecule fluorescence microscopy) is still at its infancy and would still require technological improvement to be easily and fully operational. Such progress would enlarge our vision of the biomolecular and biophysical mechanisms taking place in living cell and would help to draw the big picture of the cellular dynamics. Although it offers high spatial resolution and force sensitivity, the use of the different modes described in this Review is time consuming and not suitable for sample screening or high statistic content. Yet, several laboratories- or compagnies-have focused their efforts on the development of new modes to get rid of the slowness of the cantilever movement in traditional AFM modes. As such, high speed AFM and fast scanning modes allow now to image –with unprecedented time resolution- the dynamic of isolated molecular structures or cell surface rearrangements in real time. Recent emergence of ultrastable instruments has also enable to limit thermal drift and therefore to improve spatial resolution. Additionally, recent developments have rendered possible the acquisition of multiparametric AFM features to characterize biological samples. This new approach permits to measure simultaneously and to correlate high resolution images with the mechanical properties and adhesiveness of the sample. Although multiparametric AFM cannot always give access to single molecule unfolding (*e.g.* for long polymers or whole cell adhesion forces) due to the high velocity of the tip approach and retraction in such modes, it greatly increases the potential of AFM to address relevant biological questions.

Finally, parallel applications have also emanated which multiply the possibilities offered by AFM in biology. For instance AFM cantilevers have been used as sensors. In that sense, by monitoring cantilever fluctuation, it is now possible to weight cells and monitor their mass fluctuation under metabolic reactions, to sense mitochondrial activity or to assess bacterial resistance to antibiotics.

## Declaration of Competing Interest

The authors declare that they have no known competing financial interests or personal relationships that could have appeared to influence the work reported in this paper.
